# Heritability of territory of ruptured and unruptured intracranial aneurysms in families

**DOI:** 10.1371/journal.pone.0236714

**Published:** 2020-08-03

**Authors:** Mayte Sánchez van Kammen, Romain Bourcier, Charles J. Moomaw, Joseph P. Broderick, Daniel Woo, Chrysanthi Papagiannaki, Olivier Levrier, Antti E. Lindgren, Timo Koivisto, Juha E. Jääskeläinen, Gabriël J. E. Rinkel, Ynte M. Ruigrok

**Affiliations:** 1 Department of Neurology and Neurosurgery, University Medical Center Utrecht, Utrecht, The Netherlands; 2 Department of Neuroradiology, Nantes University Hospital, Nantes, France; 3 Department of Neurology and Rehabilitation Medicine, University of Cincinnati, Cincinnati, Ohio, United States of America; 4 Department of Neuroradiology, University Hospital of Rouen, Rouen, France; 5 Department of Neuroradiology, Hospital Clairval-Ramsay Santé, Marseille, France; 6 Department of Neurosurgery, Kuopio University Hospital, Kuopio, Finland; 7 Department of Neurosurgery, University of Eastern Finland, Kuopio, Finland; Instituto Mexicano del Seguro Social (IMSS) HGZ 2, MEXICO

## Abstract

**Background:**

A previous study suggested that intracranial aneurysms are more likely to occur in the same arterial territory within families. We aimed to replicate this analysis in independent families and in a sample limited to intracranial aneurysms that ruptured.

**Methods:**

Among families with ≥2 first-degree relatives with intracranial aneurysms, we randomly matched index families to comparison families, and compared concordance in intracranial aneurysm territory between index and comparison families using a conditional logistic events/trials model. We analyzed three European cohorts separately, and pooled the results with those of the Familial Intracranial Aneurysm study by performing an inverse variance fixed effects meta-analysis. The main analysis included both unruptured and ruptured intracranial aneurysms, and a secondary analysis only ruptured intracranial aneurysms.

**Results:**

Among 70 Dutch, 142 Finnish, and 34 French families, concordance regarding intracranial aneurysm territory was higher within families than between families, although not statistically significant. Meta-analysis revealed higher concordance in territory within families overall (odds ratio [OR] 1.7, 95%CI 1.3–2.2) and for each separate territory except the anterior cerebral artery. In the analysis of ruptured intracranial aneurysms, overall territory concordance was higher within families than between families (OR 1.8; 95%CI 1.1–2.7) but the territory-specific analysis showed statistical significance only for the internal carotid artery territory.

**Conclusions:**

We confirmed that familial intracranial aneurysms are more likely to occur in the same arterial territory within families. Moreover, we found that ruptured aneurysms were also more likely to occur in the same arterial territory within families.

## Introduction

A positive family history is the strongest risk factor for aneurysmal subarachnoid hemorrhage [1]. This suggests that genetic factors play an important role in the formation and rupture of intracranial aneurysms (IAs). The Familial Intracranial Aneurysm (FIA) study [2] has conducted an analysis of family units with two or more first-degree relatives (FDRs) affected with IAs from the United States, Canada, New Zealand, and Australia. The study reported that affected members of the same family were more likely than individuals from unrelated families to have IAs in the same arterial territory (odds ratio [OR] 1.8, 95% confidence interval [95%CI] 1.3–2.5) [3]. IA concordance was higher within family units than between family units for three of the four major arterial territories—internal carotid artery (ICA), middle cerebral artery (MCA), and vertebrobasilar system (VB)—with no difference for the anterior cerebral artery (ACA). In the FIA analysis, all IAs were included, and ruptured IAs were treated the same as unruptured IAs. We hypothesized that a high concordance in arterial territory within families may also be seen in an analysis of ruptured IAs only.

The present study sought to replicate the FIA findings in independent cohorts of Dutch, Finnish, and French families with a family history of IA and to combine the results of the three European cohorts with those of the FIA study [3] by means of a meta-analysis. In addition, we aimed to perform a secondary analysis limited to ruptured IAs to determine whether the FIA findings remain true in ruptured IAs specifically.

## Methods

### Study populations and data collection

For the Dutch families all available information from 1993 to 2015 was used from the prospectively-collected database of a consecutive series of families with familial aneurysms at the University Medical Center Utrecht, the Netherlands [4]. For the Finnish families the saccular intracranial aneurysm database of Kuopio University Hospital (KUH), Kuopio, Finland was used which includes all sporadic and familial intracranial aneurysm patients admitted to KUH between 1980 and 2017 [5]. The KUH solely serves a defined catchment population in Eastern Finland. Last, for the French families data of the ICAN project were used which is a non-interventional nationwide research program including large pedigrees with familial forms of intracranial aneurysms [6]. IRB approval was obtained for the different cohorts.

In order to optimally compare our results with those of the FIA study, we followed the previously described methods of the FIA analysis with regard to inclusion and exclusion criteria of families, definitions of affected family members, identification of probands, and definitions of arterial territories [3]. To summarize, we included affected first-degree relatives (FDRs) from all family units in which at least two FDRs had IAs. Families were excluded if they had a family history of polycystic kidney disease, Ehlers-Danlos syndrome, Marfan syndrome, Loeys-Dietz syndrome, fibromuscular dysplasia, or moya moya disease. The proband was designated as the affected family member with the highest number of affected FDRs; if there was a tie for highest number of FDRs, the member who was first brought to medical attention was designated as the proband. Aneurysm locations were retrieved from radiologic reports or medical records and classified into four major arterial territories: (1) internal carotid artery (ICA), including posterior communicating artery, ophthalmic artery, anterior choroidal artery, and distal ICA; (2) middle cerebral artery (MCA); (3) anterior cerebral artery (ACA), including anterior communicating artery and pericallosal artery; and (4) vertebrobasilar system (VB), including basilar and vertebral arteries, anterior inferior cerebellar artery, posterior inferior cerebellar artery, superior cerebellar artery, and posterior cerebral artery. As in the FIA study, IAs with unknown location were excluded. We also recorded the rupture status of each IA.

### Statistical analysis

We refrained from an a priori sample size calculation, but instead included all eligible families from each cohort of this study. Within each family, we calculated the proportion of FDRs who had IAs in the same territory as the designated proband of that family (concordance proportion). We randomly matched each index family to a comparison family, selected with replacement from the group of families whose proband had an aneurysm in a different arterial territory. We compared concordance between the index proband and the proband’s own FDRs with concordance between the index proband and the FDRs of the comparison family using the conditional logistic events/trials model previously described by the FIA study [3]. As in the FIA analysis, all IAs (ruptured and unruptured) were included. We evaluated both overall concordance as well as concordance for each of the four major arterial territories. The analysis was simulated 1001 times; we report median concordance proportions and ORs with 95% confidence intervals. The Dutch, Finnish, and French results are reported separately. Subsequently, we assessed heterogeneity between the results of the different European cohorts and the FIA study results [3]. If I^2 was <30%, we pooled the results by performing an inverse variance fixed effects meta-analysis of the median ORs with 95% CIs. In the secondary analysis we analyzed ruptured IAs only to assess the concordance in the location of these ruptured IAs within families. For this analysis we included family units that had at least two FDRs with ruptured IAs. Due to the low number of eligible family units, we pooled the Dutch, Finnish, FIA, and French families for this analysis. All analyses were performed with SAS 9.4 (SAS Institute, Cary, NC).

## Results

### Analysis on unruptured and ruptured IAs together

Of 250 eligible families, 49 were excluded because less than two family members remained after excluding those with unknown IA location, and four were excluded because of a family history of polycystic kidney disease. Thus, we included 70 Dutch, 142 Finnish, and 34 French families with familial IAs. The Dutch families included 70 probands (75.7% women) and 102 FDRs (65.7% women), the Finnish families included 142 probands (52.8% women) and 165 FDRs (49.7% women), and the French families included 34 probands (70.6% women) and 45 FDRs (71.1% women).

Table 1 compares concordance proportions between index probands and their own FDRs versus the index probands and comparison family FDRs. The concordance proportions between probands and their own FDRs were higher overall and for each arterial territory separately (except for the MCA territory in the Finnish families), although these differences did not reach statistical significance. Due to low numbers of VB IAs in the Finnish and French families, it was not possible to compare the concordance proportions for that arterial territory in these cohorts.

**Table 1:** Concordance proportions in aneurysm arterial territory in index and comparison families in the Dutch, Finnish, and French families.

	70 Dutch families			142 Finnish families			34 French families		
Arterial territory	Concordance proportions			Concordance proportions			Concordance proportions		
	Index families	Comparison families*	OR (95% CI)*	Index families	Comparison families*	OR (95% CI)*	Index families	Comparison families*	OR (95% CI)*
ICA	65.0%	40.0%	3.0 (1.0–9.2)	31.3%	21.3%	1.7 (0.6–4.5)	48.9%	33.3%	2.1 (0.5–8.8)
ACA	34.1%	26.6%	1.5 (0.5–4.3)	35.6%	28.2%	1.6 (0.7–3.6)	50.0%	9.1%	5.9 (0.5–40.3)
MCA	38.9%	28.8%	1.4 (0.4–4.3)	59.5%	60.5%	1.0 (0.5–1.7)	40.6%	35.6%	1.4 (0.4–4.9)
VB	18.8%	12.5%	2.1 (0.1–18.3)	13.3%	0%	NA	0%	0%	NA
All	47.0%	34.0%	1.8 (0.9–3.4)	52.3%	47.3%	1.3 (0.8–2.1)	54.3%	36.3%	2.4 (0.9–6.3)

ICA = internal carotid artery; ACA = anterior cerebral artery; MCA = middle cerebral artery; VB = vertebrobasilar system; OR = odds ratio; 95% CI = 95% confidence interval; NA = not applicable/unable to calculate.

* Values represent medians of 1001 iterations.

Table 2 compares the results of the present analyses with the results from the FIA study on 323 families and presents the pooled results after meta-analysis. Heterogeneity between the study cohorts was small (I^2^ range 0%–11%). The pooled results revealed that affected FDRs were more likely to have IAs in the same arterial region than individuals from unrelated families. This was evident in the overall analysis as well as the analyses on the individual arterial regions, except for the ACA territory.

**Table 2:** Meta-analysis of the results on concordance in aneurysm arterial territory within Dutch, Finnish, French, and FIA study families.

Arterial territory	70 Dutch families	142 Finnish families	34 French families	323 FIA study families [3]	Pooled results*	Heterogeneity (I^2, %)*
	OR (95% CI)	OR (95% CI)	OR (95% CI)	OR (95% CI)	OR (95% CI)	
ICA	3.0 (1.0–9.2)	1.7 (0.6–4.5)	2.1 (0.5–8.8)	1.5 (1.0–2.3)	1.7 (1.2–2.4)	0
ACA	1.5 (0.5–4.3)	1.6 (0.7–3.6)	5.9 (0.5–40.3)	1.0 (0.5–1.9)	1.3 (0.8–2.1)	0
MCA	1.4 (0.4–4.3)	1.0 (0.5–1.7)	1.4 (0.4–4.9)	2.0 (1.2–3.2)	1.5 (1.1–2.1)	11
VB	2.1 (0.1–18.3)	NA	NA	2.9 (1.1–8.2)	2.8 (1.1–7.1)	0
All	1.8 (0.9–3.4)	1.3 (0.8–2.1)	2.4 (0.9–6.3)	1.8 (1.3–2.5)	1.7 (1.3–2.2)	0

ICA = internal carotid artery; ACA = anterior cerebral artery; MCA = middle cerebral artery; VB = vertebrobasilar system; OR = odds ratio; 95% CI = 95% confidence interval; NA = not applicable/unable to calculate.

* Inverse variance fixed effects meta-analysis.

### Analysis on ruptured IAs only

We included 37 Dutch, 43 Finnish, 89 FIA, and 22 French families with ruptured IAs. The Dutch families included 37 probands (62.2% women) and 46 FDRs (71.7% women), the Finnish families included 43 probands (53.5% women) and 53 FDRs (56.7% women), the FIA families included 89 probands (64.1% women) and 101 FDRs (73.3% women), and the French families included 22 probands (68.2% women) and 24 FDRs (58.4% women).

Table 3 compares concordance proportions between index probands with ruptured IAs and their own FDRs with ruptured IAs versus the index probands and the comparison family FDRs with ruptured IAs. The concordance proportions between probands and their own FDRs were higher overall (OR 1.8; 95% CI 1.1–2.7); for the individual arterial regions, only the ICA territory reached statistical significance (OR 2.6; 95% CI 1.1–5.9). Due to low numbers of ruptured VB IAs, it was not possible to compare the concordance proportions for that category.

**Table 3:** Concordance proportions in ruptured aneurysm arterial territory in index and comparison families in the pooled Dutch, Finnish, French, and FIA families.

Arterial territory	Concordance proportions		OR (95% CI)*
	Index families	Comparison families*	
ICA (n = 51)	49.7%	29.4%	2.6 (1.1–5.9)
ACA (n = 62)	36.3%	28.2%	1.5 (0.7–3.1)
MCA (n = 59)	36.2%	23.7%	1.8 (0.8–3.9)
VB (n = 21)	0%	7.1%	NA
All (n = 191)	36.2%	25.4%	1.8 (1.1–2.7)

ICA = internal carotid artery; ACA = anterior cerebral artery; MCA = middle cerebral artery; VB = vertebrobasilar system; OR = odds ratio; 95% CI = 95% confidence interval; NA = not applicable/unable to calculate.

* Values represent medians of 1001 iterations.

## Discussion

Our findings confirm the prior report that IAs are more likely to occur in the same arterial territory within the same family. In three additional independent samples of Dutch, Finnish, and French families with aneurysms, we found effect sizes comparable to those of the original FIA study [3]. Higher concordance within families holds true overall, as well as for each major territory separately, except for the ACA territory. In addition, we found overall higher familial concordance in the analysis limited to ruptured IAs as well, and in the ICA territory specifically.

In addition to our current study and the aforementioned FIA study [3], two other studies also analyzed aneurysm territory in affected relatives. We did not include these in our meta-analysis due to different inclusion criteria and methodology. One study showed that IAs in 107 sibling pairs occurred significantly more often in the same territory compared with patient pairs randomly selected from a study of 2627 patients with sporadic IAs [7]. In the second study, the FIA study group analyzed aneurysm territory in affected twin pairs and found a higher concordance in aneurysm arterial territory in monozygotic twins, compared with dizygotic twins [8].

For our study we used similar inclusion criteria and the analysis method used by the FIA study [3] to allow for reliable comparison. In the present study, data on risk factors for IAs such as smoking or hypertension were not uniformly available. These risk factors may be possible effect mediators; MCA IAs have been found to be more associated with hypertension [9] and basilar top IAs with smoking [10] compared with IAs in other territories. Another limitation of this study is that patients with missing data on aneurysm territories could not be included, particularly among those who died from aneurysmal SAH. Consequently, as in the original FIA report, we had to exclude a considerable proportion of families due to unknown aneurysm territory and it is unclear how this information would have affected our results. Furthermore, our sample size was relatively small. Although we did not perform an a priori sample size calculation, this likely limited our ability to analyze concordance proportions for specific arterial territories. Lastly, family members might develop new IAs in the future and currently unruptured IAs might rupture, it is uncertain how this would influence our results.

The observed high familial concordance in aneurysm territory may support a genetic influence on aneurysm arterial territory. A previous study found that single nucleotide polymorphisms known to be associated with IA were correlated with IA location at the MCA [11]. Alternatively, shared environmental influences have been proposed as an important factor in the occurrence of familial subarachnoid hemorrhage [12], and these may also play a role in the observed familial concordance in aneurysm location. Future studies should concentrate on genetic and environmental risk factors predisposing to the development of aneurysms at a particular arterial territory.

## Supporting information

S1 Checklist (DOCX)

S1 File Dutch families data. (XLS)

S2 File Finnish families data. (XLS)

S3 File French families data. (XLS)

S4 File Analysis of ruptured IA data. (XLS)
