# Correction: Heritability of territory of ruptured and unruptured intracranial aneurysms in families

**DOI:** 10.1371/journal.pone.0253679

**Published:** 2021-06-17

**Authors:** The PLOS ONE Staff

The first author’s initials appear incorrectly in the citation. The publisher apologizes for the error. The correct citation is: Sánchez van Kammen M, Bourcier R, Moomaw CJ, Broderick JP, Woo D, Papagiannaki C, et al. (2020) Heritability of territory of ruptured and unruptured intracranial aneurysms in families. PLoS ONE 15(8): e0236714. https://doi.org/10.1371/journal.pone.0236714.

The first author’s initials also appear incorrectly in the Copyright statement. The publisher apologizes for the error. The correct statement is: © 2020 Sánchez van Kammen et al. This is an open access article distributed under the terms of the Creative Commons Attribution License, which permits unrestricted use, distribution, and reproduction in any medium, provided the original author and source are credited.
